# Recruitment and Retention in the National Diabetes Prevention Program Lifestyle Change Program in Two Federally Qualified Health Centers in Rural Hawaiʻi

**DOI:** 10.5888/pcd21.240156

**Published:** 2024-10-31

**Authors:** Kara Saiki, Alena Shalaby, Blythe Nett, Lance Ching, Jermy-Leigh B. Domingo, Jennifer D. Valera, Rachel Randall, L. Brooke Keliikoa, Meghan D. McGurk

**Affiliations:** 1 Healthy Hawai‘i Evaluation Team, Office of Public Health Studies, University of Hawaiʻi at Mānoa, Honolulu; 2 Department of Global Studies, University of California, Santa Barbara; 3 Hawaiʻi State Department of Health, Honolulu; 4 Hawaiʻi Primary Care Association, Honolulu; 5 CVS Caremark, Honolulu

## Abstract

Prediabetes disproportionately affects racial and ethnic minority groups in Hawai‘i. The National Diabetes Prevention Program lifestyle change program (National DPP LCP) decreases the risk of developing diabetes. However, enrolling and retaining participants is a challenge for program providers. This evaluation aimed to understand factors that influence racial and ethnic minority groups in Hawai‘i to enroll in and complete the program. From 2018 through 2023, two federally qualified health centers (FQHCs) in rural Hawai‘i administered 6 year-long cohorts. Trained lifestyle coaches, who were FQHC staff members, recruited participants and facilitated the evidence-based curriculum. In 2023, the evaluation team conducted semistructured interviews with 14 of the 40 enrolled participants (35%), all of whom were women aged 25 to 74 years. Six participants identified as Native Hawaiian or Other Pacific Islander and 3 as Filipino. Eight participants reported completing the program. We used qualitative methodology to analyze transcripts. We identified themes around motivators, barriers, facilitators, and suggestions for improvement. Recruitment by trusted individuals in their communities motivated participants to enroll. Caregiving and work obligations were attendance barriers for early withdrawers and graduates. Social support from lifestyle coaches and enrolled friends and family were facilitators for program completion. Suggestions included improving class availability and incorporating culturally relevant recipes. Barriers experienced by Native Hawaiian or Other Pacific Islander and Filipino participants were similar to those reported by racial and ethnic groups in other studies. Program providers in rural communities should use trusted individuals as lifestyle coaches and recruit family and friends, regardless of National DPP LCP eligibility, to reduce caregiving barriers and engage critical support systems to facilitate completion.

**Summary**

**What is already known on this topic?**

The National Diabetes Prevention Program lifestyle change program (National DPP LCP) prevents or delays the onset of type 2 diabetes. Native Hawaiian, Other Pacific Islander, and Filipino adults have high rates of prediabetes and low rates of enrollment in these programs.

**What is added by this report?**

The perspectives of Native Hawaiian, Other Pacific Islander, and Filipino women provide insights into how program participation among these groups can be bolstered in rural communities.

**What are the implications for public health practice?**

Having trusted members of the community help with recruitment and lead the program is effective in engaging Native Hawaiian, Other Pacific Islander, and Filipino adults. Cultural tailoring and support from family contribute to engagement and enrollment in these lifestyle change programs.

## Introduction

Prediabetes affects 38% of adults in the US (1), but only 14.9% of adults in Hawaiʻi (2). When data from Hawaiʻi are disaggregated, substantial racial and ethnic disparities exist, with Native Hawaiian (NH, 17.2%), Other Pacific Islander (OPI, 16.9%), and Filipino (17.3%) adults having higher rates than non-Hispanic White (9.0%) adults (2-4). Various behavioral, socioeconomic, and cultural reasons contribute to this disparity (4,5).

The Centers for Disease Control and Prevention (CDC) implemented the National Diabetes Prevention Program lifestyle change program (National DPP LCP) for people with prediabetes to reduce their risk of progressing to type 2 diabetes. National DPP LCP participants who were successful in making lifestyle changes have reduced their risk of progressing to diabetes by up to 58% (6). Despite the program’s benefits and the high rates of prediabetes in the US, enrolling and retaining people in the program is challenging. Less than 1% of people with prediabetes enroll in the National DPP LCP, and even fewer graduate (1,7,8); in Hawaiʻi, 1.3% of those diagnosed with prediabetes enroll (2,9).

Disparities in enrollment and retention are further evident when rates are disaggregated by race and ethnicity. US enrollment data from 2012 through 2019 identified 0.8% as NHOPI (Native Hawaiian, Other Pacific Islander), 3.1% as Asian American, and 64.6% as non-Hispanic White adults (7). Enrollment barriers include lack of program awareness, inconvenient locations, shock about their diagnosis, and feeling unmotivated or overwhelmed by other health conditions (10–12). Data suggest that people do not complete the year-long National DPP LCP because of scheduling conflicts, lack of childcare or transportation, inability to relate to other participants, dissatisfaction with the lifestyle coach, and/or class content not meeting expectations (10–12). However, these data have been mostly among non-Hispanic White, Hispanic, and Black adults (11,13). Little is known about barriers among NHOPI and Filipino adults, who are underrepresented in both enrollment and retention in the National DPP LCP (7,8). One study among NHOPI and Filipino adults examined the Partnership for Improving Lifestyle Intervention (PILI) ʻOhana Project, a culturally adapted diabetes prevention program focused on weight loss that did not meet the duration requirements of a CDC-approved program (4). That study explored barriers and facilitators encountered by NHOPI and Filipino adults in participating and completing the program, but it did not exclusively examine data for people with prediabetes. Therefore, a critical gap in the literature needs to be filled to increase enrollment and retention of NHOPI and Filipino adults in the National DPP LCP.

In 2018, the Hawaiʻi Department of Health received a 5-year grant from CDC to improve the identification of patients with prediabetes and enroll people in National DPP LCPs at federally qualified health centers (FQHCs). The evaluation focused on programs at 2 FQHCs located in rural, medically underserved areas on Hawaiʻi Island and Oʻahu (14). Hawaiʻi Island is nearly 7 times larger than Oʻahu, with many residents needing to travel long distances to access health care. The Hawaiʻi Island FQHC has 4 clinic sites located across 50 miles of coastline and serves nearly 8,000 patients (15). Most people they serve belong to racial and ethnic minority groups, one-third are Medicaid beneficiaries, and one-quarter live at or below 100% of the federal poverty level (15). Most of the Oʻahu FQHC’s nearly 5,000 patients belong to racial and ethnic minority groups, and one-half are Medicaid recipients or earn below 100% of the federal poverty level (15,16). During the 5-year grant, the Hawaiʻi Island FQHC completed 5 year-long cohorts and the Oʻahu FQHC completed 1 year-long cohort comprising employees who were diagnosed with prediabetes. Employees were recruited to pilot the program before the National DPP LCP was promoted to the patient population.

## Purpose and Objectives

The purpose of this evaluation was to understand factors influencing enrollment and retention in the National DPP LCP from the perspectives of NHOPI and Filipino participants at 2 FQHCs in rural Hawaiʻi. Funders selected these FQHCs because the FQHCs’ leadership was receptive to participating in an evaluation and because funders wanted to collect information on the perspectives of participants in an established program and a newly implemented program, which were represented by these 2 FQHCs. This process evaluation was guided by CDC’s Framework for Program Evaluation (17) and sought to gather information to help other organizations tailor their recruitment and implementation to support engagement of NHOPI and Filipino adults in rural communities.

## Intervention Approach

Participants were recruited into the National DPP LCP at each FQHC either by a referral from their health care provider or directly by lifestyle coaches, who were trusted health center staff and community members. Classes were conducted via 3 modes: exclusively in-person, exclusively virtually, or a hybrid of the 2 modalities. During classes, lifestyle coaches led participants through designated lessons by using a standard training manual and incorporated interactive components, such as local food demonstrations, group physical activities, and stress management techniques, to build participant self-efficacy to implement lifestyle changes. Lifestyle coaches tracked participant progress through weight changes and minutes of physical activity, facilitated goal setting to support lifestyle changes, and provided encouragement via text messages between classes.

## Evaluation Methods

In 2023, the University of Hawaiʻi evaluation team conducted 45-to-60–minute semistructured interviews with 14 former and current participants of the National DPP LCP via Zoom (10 participants turned their video on and 4 did not). Participants also completed an online survey that asked about age, race and ethnicity, family history of diabetes, participation modality, completion status, and familiarity with the lifestyle coach before the program. The University of Hawaiʻi Institutional Review Board designated this evaluation project as non–human subjects research, per the revised Common Rule of 2018.

### Recruitment and interview guides

The evaluation team developed interview guides in collaboration with FQHC lifestyle coaches, key partners, and the Hawaiʻi Department of Health. The semistructured interviews were used to understand participant experiences and reasons they enrolled, attended, or withdrew from the program. Questions included characteristics of their program classes and feelings about their lifestyle coach. Participants in all 6 cohorts at the 2 participating FQHCs were eligible to participate in the study, and lifestyle coaches personally reached out to their participants to assess their interest in participating in this study.

### Data analysis

Of the 40 people enrolled in the 6 cohorts, 14 agreed to be interviewed. Interviews were recorded, transcribed, and analyzed by using the “Sort and Sift, Think and Shift” qualitative data analysis methodology (18). During an initial learning period, 2 coders (K.S. and A.S.) used NVivo version 20 Pro/Plus to independently review 3 transcripts and identify themes across participants. They then discussed any divergence until reaching a consensus for each transcript. They repeated this process for all transcripts. The evaluation team summarized findings and reported them to the FQHCs, the Hawaiʻi Department of Health, and other health providers implementing the National DPP LCP. The audience appeared to accept the themes and requested future evaluations of additional programs.

## Results

Thirteen of 14 interviewed participants completed the survey (Table 1). All participants were women, and most (n = 9) were aged 25 to 44 years. Six reported being NHOPI and 3 Filipino. All but 2 participants attended classes in person. One participant attended classes exclusively virtually because the FQHC was an hour away, and the other attended hybrid classes because their cohort transitioned online during the COVID-19 pandemic.

**Table 1:** Characteristics of Survey Participants (N = 13) and Interview Participants (N = 14) and How They Experienced the National Diabetes Prevention Program (DPP) Lifestyle Change Program at Two Federally Qualified Health Centers in Rural Hawaiʻi, 2023^a^

Characteristic	No.
**Race and ethnicity^b^ (n = 13)**	
NHOPI	6
Filipino	3
Non-Hispanic White	3
Did not want to answer	1
**Age group, y (n = 13)**	
18–24	0
25–34	6
35–44	3
45–54	1
55–64	1
65–74	2
≥75	0
**Family history of diabetes (n = 13)**	
Yes	11
No	2
**Family member with diabetes^c^ (n = 11)**	
Parent	6
Grandparent	6
Sibling	3
Other family member	1
**Observed gender (n = 14)**	
Woman	14
Man	0
**Program modality experienced (n = 14)**	
In-person exclusively	12
Virtual exclusively	1
Hybrid	1
**Self-reported completion of the National DPP Lifestyle Change Program (n = 14)**	
Yes	8
No	6
**Familiar with lifestyle coach before enrollment (n = 14)**	
Yes	12
No	2
**Participant type (n = 14)**	
FQHC employee	9
Non-FQHC employee	5

Abbreviations: FQHC, federally qualified health center; NHOPI, Native Hawaiian and Other Pacific Islander.

a All 14 interviewees were asked to complete the survey after they were interviewed; 13 completed it.

b Participants were first asked to mark all race and ethnicities that applied to them, followed by the race or ethnicity that best represents them; values here are the latter.

c Eleven participants with a family history reported multiple family members with diabetes.

Eight participants reported completing the year-long cohort. The reasons participants dropped out included caregiving issues, being too busy at work, and moving out of state. Of the 6 participants who withdrew early, 4 were FQHC employees.

Interview themes were 1) motivators to enroll in the National DPP LCP, 2) barriers to participation, 3) facilitators that increased participation, and 4) suggestions to improve the program (Table 2).

**Table 2:** Barriers and Facilitators to Enrolling and Participating in the National DPP Lifestyle Change Program: Quotes From Interview Participants (N = 14) From Two Federally Qualified Health Centers in Rural Hawaiʻi, February 2023

Theme	Quote	Participant identifier
**Motivators to enroll in the National DPP lifestyle change program**		
Reaction to diagnosis	I was shocked at that time, and like that’s when I told myself I need to like change how I eat, to be better for myself and to be healthy . . . not only for myself [but also for the] people around me, like my family, friends.	Participant 13
Family history of diabetes	I was surprised, but not too surprised, only because I know how much I love my sweets. . . . But with them telling me, hey, you’re prediabetic, you gotta start doing something. It was a shock, it was like an eye opener for me. . . . And of course, seeing my dad’s situation. He’s the only one, really, in my family who had diabetes. No one else did. So, I don’t want to go through the same route that my dad did.	Participant 14
Trust in their lifestyle coach	Well, [the lifestyle coach and I] we’re friends. . . . It’s nice living in a small town, because everybody knows everybody. She had talked to me about it, and asked me if I wanted to go on this plan, and I said, “Sure, you know every little thing you can learn helps.”	Participant 1
**Barriers to participation**		
Initial feelings about the program/barrier to enrollment	When you hear something about people trying to tell you how to eat, you don’t want to hear that. It’s no, you’re going to eat whatever you want to eat. But then, after that first initial [meeting with the lifestyle coach], I thought like, “Oh, wow! This is something different, like maybe I’m gonna like it after all.”	Participant 9
Caretaking responsibilities/barrier to attendance	I had, like, a lot of things going on that I couldn’t really commit to leaving my house, and then going to, you know, the facility, and then sitting there with everybody . . . when you have to be at home with the kids, watching your parents, anything like that.	Participant 2
**Facilitators that increased participation**		
Social support from lifestyle coach	When she talks, I know she’s talking to me . . . as a friend. So, it’s a caring kinda talk, and when somebody talks to you in a caring way, you kind of more believe them.	Participant 1
Social support from cohort members	It just motivated us because we were all just doing a competition with each other, like, you know, who loses more weight? Who eats cleaner? . . . And then our favorite thing was every Wednesday we came together, and we’re like, “Guess what, guys? I’m like one pound less, or like five pounds less.”	Participant 12
**Suggestions to improve the program**		
Increasing class availability and offerings	Not just having one time available [for class]. I think that would be helpful. Instead of just having one class, I think it’d be nice if maybe you have multiple classes. Let’s see, [issues with classes at a certain] time of the day [or lack of] multiple classes. That’s just what was hard for me, personally.	Participant 14
Expanding eligibility to National DPP Lifestyle Change Program	Not just for the patients who currently have prediabetes, but like just sending it out to their families, because family . . . [may] know of other people who might be interested.	Participant 14
Providing culturally relevant content and resources	If we talked about something, and it wasn’t so localized, we always think about how we could make it. . . . I think we talked about lau lau [traditional Hawaiian dish] one time, and someone was saying…to switch it out. You just put in sweet potato, no need put the meat. . . . We always talked about local food but how we were going to make it healthier. You know our workbook would be like, just eat potatoes.	Participant 5

### Motivators to enroll in the National DPP LCP

Participants were motivated to enroll in the program to prevent progressing to diabetes; many reported a family history of diabetes and had witnessed its effect on their family members’ lives or had seen the consequences of diabetes among their FQHC patients. Nearly three-quarters were completely shocked and/or scared by their diagnosis. Even those who were not surprised by their diagnosis expressed alarm. Familiarity with and trust in the lifestyle coaches made people receptive to learning about and willing to enroll in the program. Participant success stories shared by lifestyle coaches were also motivating.

### Barriers to participation

Participants reported barriers to both enrollment and attendance. Although all interviewees had enrolled in the National DPP LCP, not all were initially highly motivated to participate. The program seemed too intrusive or overwhelming, or presented another task for their day. For 1 individual, the fear of losing autonomy over her dietary choices was an enrollment barrier, but the lifestyle coach helped her overcome those fears.

The most common barriers to attending classes were scheduling conflicts and caregiving responsibilities. Scheduling conflicts were often reported by participants who were FQHC employees because their schedule or required clinic commitments overlapped with class times. Barriers faced by the 6 women who did not complete the program included work scheduling conflicts, lack of childcare, and moving out of state.

### Facilitators that increased participation

The biggest factor facilitating both enrollment and attendance was social support from the lifestyle coach and other participants. Lifestyle coaches’ support and confidence in participants’ ability to make behavior changes bolstered participation. Lifestyle coaches also provided make-up classes, sometimes one-on-one, to help retain participants who were unable to attend scheduled classes.

Most participants felt that the group dynamic provided them with peer support and accountability, which helped them to continue attending and striving for their health goals. They valued having a space to discuss ways to improve their diet and/or their fitness plans by sharing what had and had not worked for them. Other facilitators included having tools such as step counters and social media to track and share their progress in meeting goals.

### Suggestions to improve the program

Participants recommended offering more classes at different times and allowing family members to attend regardless of their diabetes status. They suggested holding classes in a private space to help participants feel comfortable. Tailoring the nutrition content from the standardized workbook recipes to healthier versions of culturally relevant recipes allowed participants to further engage in lessons.

## Implications for Public Health

The main barriers experienced by our sample of majority NHOPI and Filipino participants were similar to those reported in studies of other racial and ethnic populations participating in the National DPP LCP. Scheduling conflicts were the most reported barrier in studies of non-Hispanic White and Hispanic adults (10,11) and remained so for NHOPI and Filipino adults. Even with leadership support and a work culture that prioritizes health — factors that facilitate employee participation in LCPs — FQHC employees encountered difficulties attending classes held at their worksite. More evening and weekend classes would help to reduce participation barriers and were recommended by other studies (11,19). However, offering more classes poses a financial challenge for the National DPP LCP sites in terms of hiring additional lifestyle coaches and having areas to offer classes in facilities with limited space.

NHOPI and Filipino interviewees in our study reported that lack of childcare and other caregiving responsibilities interfered with their ability to participate, in alignment with other studies (19,20). To alleviate caregiver barriers, participants suggested including family members regardless of their prediabetes status and expanding eligibility criteria to include children. Literature shows that participating in the National DPP LCP with a household member can increase engagement, suggesting that including family members in classes can address caregiving barriers and increase social support to bolster program retention (21,22). Additionally, because many interviewees had a family history of diabetes, and Asian and NHOPI people are more likely than non-Hispanic White people to live in multigenerational households (23), a family-centered approach to LCPs could produce a generational effect on diabetes.

Despite barriers, effective recruitment of NHOPI and Filipino adults to the National DPP LCP in these rural communities is possible. These rural FQHCs addressed transportation barriers by offering the program virtually, similar to what was recommended in other studies (19). Promoting the program through community FQHCs and using trusted community members (eg, community health workers, FQHC employees) to conduct classes were effective strategies for recruiting these populations. Establishing community relationships is key to improving engagement of NHOPI and Filipino people in National DPP LCPs. Data from the PILI ʻOhana Lifestyle Project showed that partnerships with trusted community organizations dedicated to serving NHOPI people facilitated enrollment of racial and ethnic minority adults (4).

Our evaluation study had several limitations. First, the evaluation sample was small. Despite the low response rate, the sample was demographically similar to all who participated in the 2 FQHC programs in terms of gender (100% vs 92.5% women, respectively) and race (64.3% NHOPI and Asian vs 72.5%, respectively). Second, the sample mostly comprised FQHC employees, which may limit the generalizability of findings to other National DPP LCP sites. However, it is not unique for employees to participate in a diabetes prevention program held at their worksite (24). Third, lifestyle coaches recruited participants to the study, which may have resulted in more participation from people who had positive feelings about their experience than from people who had negative feelings. Fourth, the evaluation lacks the perspectives of participants who were referred to the program but did not enroll, which is critical to understanding barriers to enrollment. Fifth, because this evaluation occurred 4 years after the first cohort, participants in the earlier cohorts may have had limited recall of their experiences in the program. Despite these limitations, a strength of this study was that it documented the perspectives of ethnically diverse participants who were from rural communities and included perspectives of both those who completed the program and those who withdrew early. Most importantly, most participants were NHOPI or Filipino, which contributes new information on the experiences of groups that are underrepresented in research and disproportionately affected by diabetes.

Overall, our study found that barriers and facilitators experienced by NHOPI and Filipino people are similar to those experienced by people of other races and ethnicities and people in rural communities. Addressing attendance barriers through expanded class times and engaging whole families could improve engagement and retention not only of these populations, but other racial and ethnic groups as well. Our study showed that NHOPI and Filipino adults can be successfully enrolled and retained in the National DPP LCP through cultural tailoring of the curriculum and emphasizing support from trusted community members and families. These strategies can be applied to other organizations looking to enroll and retain NHOPI and Filipino populations in the National DPP LCP to reduce disparities in prediabetes and diabetes rates.
